# Why is quaternary prevention important in prevention?

**DOI:** 10.11606/S1518-8787.2017051000041

**Published:** 2017-11-16

**Authors:** Charles Dalcanale Tesser

**Affiliations:** I Universidade Federal de Santa Catarina. Centro de Ciências da Saúde. Departamento de Saúde Pública. Florianópolis, SC, Brasil

**Keywords:** Unnecessary Procedures, Quaternary Prevention, Preventive Medicine, Disease Prevention, Procedimentos Desnecessários, Prevenção de Doenças, Medicina Preventiva, Prevenção Quaternária

## Abstract

Quaternary prevention consists in the identification of persons at risk of excessive medicalization and their protection against new unnecessary interventions, avoiding iatrogenic damages. Here, we argue about the importance of quaternary prevention in specific primary and secondary prevention. The recent great development of preventive medicine, biomedicalization of risks and their treatment as if they were diseases, and the powerful influence of the commercial interests of pharmaceutical industries on the production of medical-sanitary knowledge alter classifications, create diseases and pre-diseases, lower cutoff points, and erase the distinction between prevention and healing. This situation converts larger amounts of asymptomatic persons into sick individuals and diverts clinical attention and resources from sick persons to the healthy, from older adults to young persons, and from the poor to the rich. Quaternary prevention facilitates and induces the development and systematization of operational knowledge and guidelines to contain hypermedicalization and the damages of preventive actions in professional care, especially in primary health care.

## RESUMO

A prevenção quaternária consiste na identificação de pessoas em risco de medicalização excessiva e sua proteção contra novas intervenções desnecessárias, evitando danos iatrogênicos. Aqui, argumentamos sobre a importância da prevenção quaternária na prevenção primária específica e secundária. O grande desenvolvimento recente da medicina preventiva, da biomedicalização dos riscos e seu tratamento como se fossem doenças e a influência poderosa dos interesses comerciais das indústrias farmacêuticas sobre a produção de conhecimento médico-sanitário alteram classificações, criam doenças e pré-doenças, rebaixam pontos de corte e apagam a distinção entre prevenção e cura. Isso converte maiores proporções de pessoas assintomáticas em doentes e desvia a atenção clínica e os recursos dos mais doentes para os saudáveis, dos idosos para os jovens e dos pobres para os ricos. A prevenção quaternária facilita e induz o desenvolvimento e sistematização de saberes e diretrizes operacionais para a contenção da hipermedicalização e dos danos das ações preventivas no cuidado profissional, sobretudo na atenção primária à saúde.

## INTRODUCTION

Quaternary prevention (P4) is a relatively recent concept (and practice), which means the identification of persons at risk of excessive medicalization and their protection from further unnecessary interventions, avoiding iatrogenic damages and proposing ethically acceptable measures^18^. Other meanings have been proposed for this expression, synthesized in Starfield et al.^29^, and a previous discussion about its importance for the Brazilian Unified Health System (SUS) can be found in Norman and Tesser^22^.

The objective of this article is to present the relevance of P4 specifically for primary and secondary prevention activities^19^. We start with a synoptic commentary on preventive medicine and the types of prevention, in which P4 has appeared and has been developing. Next, we detail its relevance and meaning. Then, we discuss the preventive change in biomedicine and some of its problematic characteristics, which, as our central hypothesis, intensely demand P4.

### Preventive Medicine: Past and Present

Preventive behaviors have always existed and followed the history of health care and sickness practices in societies, including contemporary Western medicine or biomedicine. However, what we now call preventive medicine began in the first half of the twentieth century, having more presence in its second half. It consisted of a movement to construct a preventive attitude to influence medical professionals, who were then accused of focusing on curativistic actions, that is the diagnosis and cure of diseases. Preventive medicine was characterized by three premises: (1) focus on the individual and the family, (2) performed in the daily practice of physicians, (3) “represents a major transformation in the medical practice [...] and is based on the development of a new attitude by the physician” (Arouca^2^, p.12).

The fundamental theoretical basis of this movement was the model of the natural history of disease^19^, built from an ideology in which illness would arise from three main interacting factors: the etiological agent, the host, and the “environment”. The first two came from the unicausal conception derived from infectious diseases of the late nineteenth century, still very influential. Under the label of “environment”, all social, cultural, economic, environmental, etc., factors and influences were concentrated, mixed, and naturalized. The classification of Leavell and Clark^19^ of preventive actions into primary, secondary, and tertiary was widely disseminated in the medical and sanitary knowledge, and prevention had a great recent penetration in the clinical activity^29^.

The ‘natural history of diseases’ is a relative concept that should not be generalized today beyond some infectious diseases, since it was developed around them in the nineteenth century. Its reality is dubious even in many infectious processes (influenza, leishmaniasis, tuberculosis, dengue, or leprosy) and also in lupus erythematosus, schizophrenia, diabetes, lumbar disc hernia, dementia, depression, and breast cancer^11^. “Examples of diseases, abnormalities, and dysfunctions diagnosed by progressively more sophisticated methods with little or no correlation with clinical symptoms or morbid outcomes are increasingly numerous” (Tesser^33^, p.6), that is, diseases whose natural history is not known, or perhaps simply do not exist, and that are not satisfactorily explained by biomedical models. Authors have called this phenomenon “reservoir of diseases or pseudo-diseases”^34^, which is centrally involved in the complex phenomenon of overdiagnosis and which has recently been much discussed^16^, generated on a large scale by screening. The idea of the natural history of disease seems increasingly precarious in many situations, but it still remains widely used.

If the preventive medicine movement is not evaluated as successful in the twentieth century, today the incorporation of preventive actions in medical practice has advanced greatly, especially from the development of preventive pharmacological treatments, whose most famous example may be statins, for the reduction of cholesterol levels^1^.

### On Quaternary Prevention

Quaternary prevention focuses on all clinical and health activities, including other types of prevention. It concerns the necessary self-containment of this activity, now notoriously known to be a significant health risk^22^.

Leavell and Clark^19^ have organized prevention in a chronological, linear, and technical way, aiming at preventing future morbid events with actions in the present, based on medical-scientific knowledge. The expansion of the concept and practice of P4 allows and facilitates the change of this logic, based only on biomedical knowledge and time, into another one based on the relationship between professional and user. The Figure illustrates the three classic types of prevention and P4 seen from two axes: the user experience and the professional perspective.

**Figure:**
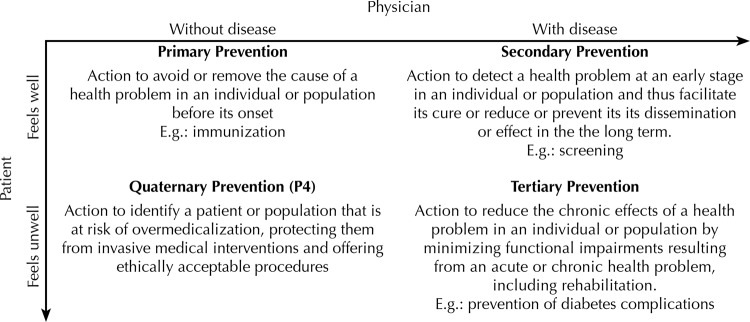
Types of preventive actions.

Source: adapted from Jamoulle^18^.

Quaternary prevention induces critical evaluation and reaction in physicians, health professionals, and professionals and managers of health systems about themselves and their activity, in an operational manner, including the questioning of their technical and ethical limits and the recognition of influences that affect decisions and preventive behaviors. It points to the construction of good practices, against cultural, technical, and institutional tendencies, which are sometimes harmful to individual and collective health. Unlike primary, secondary, and tertiary prevention, the objective of P4 is the action of professionals and health systems, especially primary health care (PHC) professionals, who have the same origin as P4^22^.

Although P4 seems to repeat the old medicine principle “*primum non nocere*”, it is relatively novel, since this principle has always referred to an ethics that was only spoken and generically proclaimed^28^ but usually relegated to the intimacy of each professional. It addresses the practical, professional, and technical development of critiques and knowledge about the process of medicalization of society, care, and prevention elaborated decades ago by authors such as Foucault^9^, Illich^17^, Zola^35^, Skrabanek^26^ ^,^ ^27^, and Clarke^7^. Although there is no conceptual novelty in it, its focus on practices is relevant and innovative. With this focus, we can move beyond its limits and contradictions, such as the adoption of nomenclature similar to that used in the model of Leavell and Clarke^19^, which could theoretically mean its reinforcement. Quaternary prevention could be defined as the practical or technical actions and developments of ethical, attitudinal, epistemological, and political resistance against the excesses of iatrogeny, preventivism, and medicalization in professional and institutional practices. Because it is more synthetic and native to PHC, the expression P4 facilitates communication with professionals and managers, communicates the emotional appeal of prevention in general, and introduces the critique in the preventive technical discussion, and therefore deserves to be maintained.

Quaternary prevention in prevention induces an organized discussion and a collective recognition of professionals and the health system about problematic situations, in which interventionist and overmedicalizing attitudes, potentially more harmful, are common, which demand guiding knowledge for containment and correction. This opens space for the construction of guidelines that need to be systematized to better protect users from the damages and risks produced by preventive clinical-health action.

### The Preventive Change

Among other factors, the therapeutic limits and the adverse effects of biomedicine for chronic diseases associated with their increasing costs and social and cultural changes that constitute a health paradox have paved the way for the emergence of a preventive change in biomedicine.

Barski^4^ has called as “health paradox” the situation in which persons subjectively feel sicker although health has improved in high-income countries. This has been attributed to some factors: reduction of mortality from infectious diseases, which generated an increase in the prevalence of chronic diseases; greater self-scrutiny regarding health, increasing attention given to the symptoms and feelings of malaise; commercialization of health and its growing presence in the media, creating an atmosphere of apprehension and insecurity about diseases and risk factors; and progressive medicalization of daily life, which has brought unrealistic expectations of healing and prevention and made intractable diseases, risks, and malaise seem even worse^4^. Medicalization has become an intense biomedicalization of life, health care, and risk (prevention)^7^, potentially converting all persons into patients^27^.

The biomedicalization and management of prevention and treatment of isolated individual risks as chronic diseases operate within a cultural industry in which there is an obsessive search for present and future health, becoming a moral obligation, value, and fashion. It puts pressure on citizens to submit to biomedical knowledges, linked to the consumption of products, services, and technologies, in addition to holding them accountable for behavioral changes that are often difficult or even unfeasible^6^ ^,^ ^24^.

In this context, the operational, conceptual, and methodological aspects of preventive medicine were made possible and driven by the great development of the production of knowledge centered on the notion of risk, especially the so-called clinical epidemiology or evidence-based medicine. This evidence-based medicine strongly diverted prevention to interventions on individuals and their risks. There was an intense “predominance of the individual over the collective, from the technical to the political, from the natural to the social, from the care physician to the sanitary physician, from the private to the public” (Ayres^3^, p.236).

The identification (and treatment) of risk factors as part of prevention began a new era in public health, focused on individual intervention. The definitions of disease have been changing over time, becoming more inclusive, with lower diagnostic thresholds. In addition, risk factors are being managed in clinical practice as if they were diseases (chronic diseases, generally). In this process, the difference between prevention and cure is becoming increasingly blurred^29^ and prevention progressively widens its scope in the clinical-biomedical action. This occurs, among other ways, from the uncritical incorporation of the “high-risk” in diseases, with serious consequences that aggravate the medicalization, interventionism, and damages of the clinical-sanitary action – which turns (conventionally, but practically) a great number of healthy persons into sick ones and generates interventions in asymptomatic individuals, all biomedicalized and exposed to greater potential for damage^32^.

Medical practice in the twenty-first century has been changing, broadly incorporating preventive attitudes and actions. Individual preventive interventions are being disseminated, based on professional and institutional clinical decisions, especially in PHC. Preventive guidelines invade medical knowledge and practice, as well as clinical guidelines and institutional norms and regulations^10^.

In the production of biomedical knowledge, the maneuvers to lower cutoff points for diagnoses of increased risk have intensified and legitimized this process. The expansion of the number of risk factors expands the area of action of preventive intervention, also expanding the number of potentially treatable situations. Specifically, this means the increasingly early biomedicalization and intervention in “pre-disease” states and risk factors, with ever more rigid and difficult-to-reach targets requiring the use of medications. This occurs, for example, with hypertension, hypercholesterolemia, obesity, and osteopenia. The consequent commercialization of drugs for asymptomatic persons substantially contributes with the expansion of these markets and increases polypharmacy, especially in older adults, generating increased iatrogenesis. In addition, it increases costs for society and health services. Finally, this increasingly common and present trend can reduce the quality of life by converting healthy persons into chronic patients^29^.

With the progressive elimination of the distinction between prevention and cure, the generation of pseudo-diseases and “pre-diseases”, and the medicalization of risks, preventive and therapeutic clinical demands are created for national health systems and medical practice, especially in PHC, being these demands largely not considered as medical problems in the past and mostly disconnected from the feeling of sickness. In addition, preventive actions have been indicated without adequate foundation, being accumulated in professional practices and, consequently, increasing the chance of iatrogenic damages^12^ ^,^ ^14^.

As a psychological, cultural, and technical consequence, users (healthy and sick ones) and professionals have progressively less tolerance to the oscillations and variations of the individual health and disease process. This lower tolerance produces demand and pressure for increasingly early interventions. Thus, we have a decrease in the margin of normality and an increase in the pathological and higher risk spectrum, this one managed as a disease. Therefore, more diagnoses and preventive actions are carried out, with complementary tests and drugs; and the interval of safety, the margin between benefits and risks, decreases. “Patients are increasingly being treated with a greater degree of diagnostic and therapeutic resources” (Gérvas^10^, p. 129), thus increasing the likelihood of iatrogenic damage. Starfield et al.^2^ ^9^ have even claim that the practice of medicine, and particularly the practice of prevention in medicine, is increasingly distancing itself from its historical and social roots of care focused on truly sick persons.

Another important factor reinforces this biomedicalized social and technical dynamics: the linking between biomedical research studies, in the production of technologies, drugs, and clinical knowledge, and the economic interests of pharmaceutical and medical equipment and supply industries. This generated what has been called disease mongering, that is, trafficking or commodification of diseases. It addresses the manipulation of the production of specialized medical and scientific knowledge and the social, individual, and professional perception of the fluid limits between health and disease, shifting these limits to the expansion of what can be felt or interpreted and treated as disease^21^. Such manipulation, which is relatively easy to be carried out because of the conventional nature of the establishment of cutoff points in the continuum of risk and severity of situations, physiological parameters, and criteria defining diseases and risks, occurs in the sense that it increases the pathological situation, including the increased risk within it^32^. This situation expands the market of pharmaceutical companies, reduces the limits of what is normal and of low risk, and ultimately leads to the legitimization of the diagnosis of increased risks and their treatment with drugs, as if they were diseases, which closes the vicious circle that transforms the citizen into a chronic patient. Examples are many and they include: mild and moderate types of depression and anxiety, attention deficit hyperactivity disorder (ADHD), social phobia, intermittent explosive disorder, irritable bowel syndrome, restless legs syndrome, osteoporosis, hypercholesterolemia, pre-diabetes, pre-hypertension, premature ejaculation, erectile dysfunction, female sexual dysfunction, male menopause, etc.^8^

The entire process creates unjustified concern and unnecessary use of medical services and technologies, generates waste of resources in trivial situations or isolated risk factors at the expense of the care of those significantly and more seriously ill^14^, and exposes patients to iatrogenic risks. Excessive use of drugs and other medical technologies, in turn, makes disease mongering a significant public health problem, which demands P4.

Finally, the preventivist idea of the apology of individual prevention of the twentieth century was, paradoxically, renewed and intensified in the twenty-first century with the new discourses of health promotion, a relation too complex for analysis in this space but which deserve brief comments. Since the First International Conference on Health Promotion in 1986, an international movement for the revaluation of health promotion has been launched that has emancipated it from prevention and has sought to overcome its focus on disease by transforming it into an umbrella speech for health improvement. However, there are internal disputes and currents in the discourse of promotion, notably an individualistic and behaviorist approach, powerful and focused on the induction of individual healthy lifestyles, in a tension contradictory to the critical discussion and action on the social determination of the health and disease process^30^. There seems to be an association between several factors, among which we can mention the convergence between healthy and preventive behaviors, the social and symbolic force of the biomedical clinical care and its behavioral individualism, and the synergy of this individualism with the media and commercial interests related to prevention, which contributed with the confusion between prevention and promotion in clinical practice and sanitary institutions, in the social and professional imaginary, reinforcing the naive apology of its necessity at all costs^8^. For example, Buss and Carvalho^5^ affirm that actions carried out by family health teams, such as child growth and development control, immunization schedules, prenatal monitoring, stimulation of breastfeeding, and promotion of better home and individual hygiene are health promotion actions. Preventive practices that are confused or mixed with promotion feedback an individualistic preventive discourse and they may even be hindering the access of real patients to health services^29^.

It is ethically questionable and inequitable to give priority to the care of healthy persons of risk instead of those noticeably sick^15^ ^,^ ^23^. Such a process causes additional self-feedback difficulties for national public health systems and their professionals, especially in PHC. This gives rise to the paradoxical situation in which noticeably sick persons often suffer from difficulties in accessing clinical care, whereas asymptomatic persons of greater risk occupy an increasing space in health services. It is often believed that the latter ones will get sick in the future if not prioritized in the present. On behalf of the noble cause of preventing future diseases, currently sick persons are relatively sacrificed. Health systems and care practices are turning to individual prevention based on risk factors, and thus shifting resources from the poor to the wealthy, from the sick to the healthy, and from older adults to young persons^15^. Starfield et al.^29^ question whether it is justifiable for check-up appointments to be nearly half of the visits to health services in the United States, where many persons lack medical care.

Heath^16^ highlights four serious ethical implications of overdiagnosis that are extensible to the current hyperpreventivism and disease mongering: (1) the extent of the damage to many individuals, by labeling them as of risk or with a disease, which can generate fear and undermine their health and well-being; (2) the direct relationship between overdiagnosis and underdiagnosis, because whenever a diagnosis is extended, attention and resources are inevitably redirected far from the more severely affected patients; (3) the potential of making health systems unfeasible based on social solidarity, because of the increasing costs involved; and (4) the marginalization and obscuring of the socioeconomic causes of health problems promoted by hypertrophied biotechnical activity.

In addition, more risk information increases the sense of control over the lives and quality of life of individuals. However, this information can, and often does, cast shadows of doubt and insecurity on these persons and thereby undermine their experience of personal integrity, safety, and health. The more preventive initiatives emphasize risk and instruct individuals on the many ways of dying, the more uncertain and fearful the future may seem^13^. By conveying the notion of risk to users, health professionals may be “spilling a drop of ink in the clear water of their identities, which may no longer be cleared” (Sweeney^31^, p.222). Getz et al.^14^ have drawn attention to our limited understanding of the effect of being labeled as of risk or high risk.

## FINAL CONSIDERATIONS

Quaternary prevention can be considered as a native concept of PHC professionals, which synthesizes its recognition of the advance of biomedicalization and industrial and commercial colonization of clinical care, public health, and production of biomedical knowledge. Its practice implies active resistance, prudent skepticism, and generation of knowledge and practices aimed at the defense and protection of citizens and patients. It is a developing strategy to discuss, qualify, and redirect medical and health activities in order to avoid over-medicalization and iatrogenic damages^18^.

The preventive change, the disease mongering, and the influence of the knowledge industry^20^ on the production and dissemination of medical knowledge fuel the proliferation of preventive actions absorbed in medical practice, and today require a general change of posture in both health professionals and managers of health systems. The change is aligned towards P4 and includes a greater critical spirit, greater skepticism about preventive interventionism, and greater resistance against preventive measures that can trigger cascades of intervention. More ethical and technical rigor is needed in the analysis of the arguments and reasons for incorporating or recommending individual preventive measures^32^ of specific primary and secondary prevention.

Quaternary prevention in prevention has an advantage over it in clinical care: it usually does not demand the creation of alternatives. We only need not to carry out or indicate dubious or inappropriate actions, resisting the strong preventivist pressures of the current culture. This lack of action, in individual care, requires communicative skills, much empathy, and additional qualifying care work.

If they used to be a noble cause that deserved general support, today we know that individual preventive actions carry known and unknown risks and they deserve to be differentiated, with detailed study and great exigency in the ethical suitability (absence of conflicts of interest) and quality of the evidences in relation to their final results, to evaluate the balance between its damages and benefits^31^. If an unconcerned optimism and commitment in supporting individual primary and secondary prevention could happen in the twentieth century, we have the prudent skepticism typical of P4 in the twenty-first century, which requires the development and gathering of critical knowledge, greater requirement for transparency and analysis of results in the evaluation of what deserves or not to be indicated and performed, and greater resistance to emotional pressures and economic interests for the adoption of individual preventive interventions with potential risk and overmedicalization^32^.

Quaternary prevention in prevention induces the production, systematization, and collectivization of critical knowledge, strict technical criteria, and careful ethical requirements by guiding professional and institutional preventive actions. It can contribute to prevent the excessive medicalization of prevention and reduce its damages, several not being perceived by users and professionals.
